# Contextual modulation of medial prefrontal cortex to neutral faces in anxious adolescents

**DOI:** 10.1186/2045-5380-3-18

**Published:** 2013-11-15

**Authors:** Tara S Peris, Adriana Galván

**Affiliations:** 1 Division of Child and Adolescent Psychiatry, UCLA Semel Institute for Neuroscience and Human Behavior, 760 Westwood Plaza, Room 67-439, Los Angeles, CA 90095, USA; 2 Department of Psychology, University of California, Los Angeles, USA; 3 Brain Research Institute, University of California, Los Angeles, USA

**Keywords:** Anxiety disorders, Adolescence, FMRI

## Abstract

**Background:**

Although interpretation biases are well documented among youth with anxiety disorders, understanding of their neural correlates is limited. In particular, there has been little study of how anxious youth neurobiologically represent changing contextual cues that may trigger anxiety. This study examined neural responses during a task in which participants viewed neutral faces paired with experimentally manipulated contextual stimuli.

**Methods:**

Participants (16 youth with a primary anxiety disorder diagnosis and 15 age- and gender-matched controls) passively viewed neutral faces that were paired with either neutral descriptive vignettes or with vignettes that were potentially anxiety provoking (for example, those that involved performance/social evaluation).

**Results:**

The two groups were differentiated by their medial prefrontal cortex (mPFC) responses, such that context modulated mPFC activation in anxious youth while non-anxious youth demonstrated no such differentiation. Counter to expectations, the performance/evaluation frames were not associated with amygdala reactivity for either group.

**Conclusions:**

The present investigation is among the first to identify how context modulates mPFC responding to neutral stimuli among anxious youth. It takes an important step toward understanding the neurobiological correlates underlying interpretation biases of neutral stimuli in this population.

## Background

Anxiety disorders occur frequently in children and adolescents [1] affecting up to 25% of the youth population [2-4]. Characterized by marked distress and functional impairment in the short-term, anxiety disorders can derail the normal developmental trajectory and place youth at risk for a host of poor outcomes over the long term. Indeed, when left untreated, youth with these conditions are at risk for diminished school performance [5], compromised family functioning [6], and increased rates of psychiatric disorder in adulthood [7]. These risks constitute a significant public health burden, and they underscore the importance of continued efforts to understand the etiology and course of youth anxiety.

Information-processing models provide one strategy for understanding how anxiety emerges and is maintained over time. These models emphasize biases in how youth attend to, process, and interpret potentially threatening information as central to anxiety, and they have received considerable empirical support [8,9]. Research using traditional descriptive and experimental paradigms has found that anxious youth are apt to interpret neutral or ambiguous information as threatening [8-10]. These cognitive biases are thought to fuel the distress and avoidance behavior that characterize anxiety disorders, and they are viewed as potential explanatory mechanisms for understanding their etiology [10,11]. However, the neural correlates of biased interpretations of neutral stimuli in these youth remain relatively sparse. In particular, the neural locus that transforms neutral information as ‘threatening’ in anxious youth remains unknown. A candidate brain region that may underlie this phenomenon is the medial prefrontal cortex (mPFC). The mPFC has garnered substantial interest in the adolescent literature because of its role in self-concept and mentalizing [12-14], its engagement in social and emotional processes [15,16], and its protracted development throughout childhood and adolescence [17]. There is also some evidence that the ventromedial PFC is associated with trait anxiety [18] and that the ventrolateral PFC in anxious youth is hyper-responsive to fear states when viewing emotional faces [19]. Medial regions of the PFC exhibit increased activation in anxious versus non-anxious youth in response to emotional faces [20] and during viewing of others’ opinions [21]. In particular, the ventromedial PFC has been implicated in evaluative functions associated with affective processing [22] while dorsal regions of the medial PFC have been linked to appraisal of emotions [23]. These converging studies suggest that activation in mPFC is significantly implicated in anxiety.

There is to our knowledge little study of how anxious youth respond to changing contextual cues that may trigger anxiety. Research suggests that neural responses to threat are sensitive to subtle differences in context [24]. In particular, in a study examining amygdala-PFC linkages in response to surprised faces, Kim et al. [24] presented non-anxious adults with surprised faces that were preceded by vignettes that provided either a positive or negative context (for example, he just found/lost $500 dollars). They found that faces preceded by negative contextual frames were associated with increased amygdala and ventrolateral PFC activation relative to those preceded by positive frames while comparisons involving positive *versus* negative frames produced greater activation within the ventromedial PFC [24].

Based on studies suggesting heightened mPFC activation in anxious youth [18,20,21], the present investigation evaluated neurodevelopmental features of mPFC response during contextual modulation of neutral faces in anxious and non-anxious youth. These faces were paired with both descriptively neutral vignettes (‘He is watching a presentation’) and those that were potentially anxiety-provoking (‘He is about to give a presentation’). We hypothesized that: (1) the mPFC and amygdala would discriminate neutral faces based on context; and (2) that this response would be greater for anxious youth compared to healthy controls.

## Method

### Participants

Thirty-two adolescents participated (Table 1). Participants in the anxious group (ANX; *n*=16; mean age, 13.05 years; 6 girls), were required to meet Diagnostic and Statistical Manual of Mental Disorders-Fourth Edition (DSM-IV) [25] criteria for a current primary diagnosis of separation anxiety, social phobia, or GAD. Co-morbidity among these diagnoses was permitted; however, participants were excluded from participation if they met criteria for any other DSM-IV diagnosis (for example, major depressive disorder). All youth in the ANX arm were treatment-seeking, and they were excluded if they had a prior history of cognitive behavior therapy or pharmacotherapy. The healthy controls (HC) (*n*=15; mean age, 13.69 years; 5 girls) did not meet criteria for any current or lifetime DSM-IV diagnosis. Adolescents in both conditions were excluded if they were currently taking psychotropic medication, had a positive pregnancy test, endorsed current drug or alcohol abuse, or met criteria for MRI restrictions. One HC participant was excluded for excess motion.

**Table 1:** Demographic characteristics

	**Anxious group**	**Healthy control group**
	***M (SD)***	***M (SD)***
*n*	16 (6 girls)	15 (5 girls)
Age	13.05 (2.87)	13.69 (2.28)
IQ	110.35 (13.39)	100.46 (14.77)
MASC^a^		
Total score	58.81 (9.53)^b^	47.53 (10.38)^b^
Harm avoidance	59.87 (8.00)^b^	52.00 (12.38)^b^
Social anxiety	58.50 (10.83)^b^	49.53 (8.16)^b^
Physical symptoms	48.68 (9.22)	42.61 (7.95)

^a^Multidimensional anxiety scale for children.

^b^Significant group differences *P* <0.05.

### Procedure

Prior to conducting the study, written informed consent was obtained from the parents of participants, and written assent was obtained from the participants. This study was conducted approved by the UCLA Institutional Review Board (#11-002606). Participants were recruited via advertisements and flyers and direct calls from families to a pediatric anxiety specialty program at a large, academic medical center. Interested families completed an initial telephone screening to assess preliminary eligibility. They were then invited to the laboratory for two separate visits. At the first visit, participants provided written informed consent/assent and were diagnosed using the Anxiety Disorders Interview Schedule-4^th^ edition, [26], a widely-used semi-structured clinical interview for diagnosing pediatric anxiety [24], by a licensed clinical psychologist. They also completed the Wecshler Abbreviated Scale of Intelligence [27] as a measure of IQ. Participants were also acclimated to the scanner environment with a mock scanner. At visit two, participants received a brain scan. Prior to the scan, participants were given verbal and written instructions about the task. To ensure that the youngest children understood the instructions, they were read to all participants by an experimenter. After completion of the experiment participants were given monetary compensation.

### fMRI task

**Figure 1:**
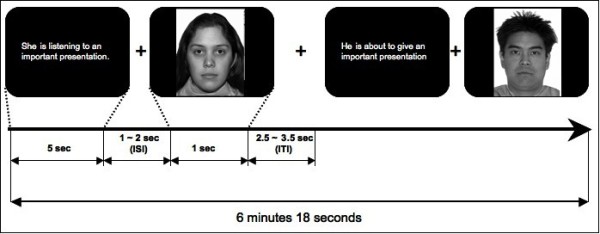
**Schematic of the task.** Participants were first presented with a contextual vignette that was followed by a neutral face.

Participants performed a modified version of a task [24], in which a face was preceded by a vignette that was intended to modulate contextual interpretation of the face. On each trial, presentation of a neutral face (1 s) was preceded by a vignette (5 s) that described either a socially neutral situation (for example, ‘She is listening to an important presentation’) or the same situation with a potentially anxiety-provoking component (for example, ‘She is about to give an important presentation’) (Figure 1). To maintain attention, they were asked to press one button if the face was male and a different button if the face was female. There were two runs (*n*=36 trials/run; 6 min 18 s), and 12 vignette-face pairs presented per run. There were two versions of the task. In Version A, faces 1 to 6 were always paired with an anxiety-provoking vignette and faces 7 to 12 were always paired with a neutral vignette. In Version B, the opposite pairing was presented. Vignette-face pairs were never presented more than once per run. Versions A and B were counterbalanced across participants. Eprime software [28] was used to generate stimuli and to collect responses. Stimuli were visualized through MRI-compatible goggles.

After the task, participants were presented with each of the faces and vignettes and asked to rate how anxious/nervous they felt on a Likert scale based on the following: ‘Imagine being in the situation described or recall a situation in which you witnessed someone else in that situation and indicate how anxious/nervous the face/vignette made you feel’. During scanning, participants responded by pressing either of two buttons on a button-response box with the pointer and middle fingers of their right hand. During the rating component of the scan, they used all four buttons of the response box.

### Stimuli and apparatus

The neutral face stimuli were taken from the NimStim Set of Facial Expressions [29]. This stimuli set is available in the public domain and was developed using actors of various genders and races who were asked to portray a range of facial expressions (for example, fearful, happy, sad, neutral) [29]. Male and female faces were counterbalanced with vignette pairing to ensure that half of each of the anxiety-provoking and neutral vignettes were paired with female faces.

The following criteria were used to generate the vignettes used in this study: (1) vignettes described common, everyday situations (for example, school, softball field, interaction with another person); (2) anxiety-provoking conditions included potential evaluation by peers, colleagues, or superiors or the potential for personal evaluation of performance; and (3) neutral conditions were as similar to anxiety-provoking conditions as possible but involved more passive observation of the target situation (that is, watching a presentation *versus* giving a presentation). Sentences in the two conditions were openly pilot tested on an initial case series of youth.

### Measures

#### Anxiety disorders interview schedule-fourth edition (ADIS-IV)

The ADIS-IV [30] is a semi-structured diagnostic interview that assesses the major DSM-IV anxiety, mood, and externalizing disorders [30]. The instrument also assigns clinical severity ratings (CSRs) following an 8-point scale (0= not at all, 8= very, very much) for each diagnosis; scores of four or higher indicate a clinically significant anxiety disorder. The ADIS-IV has well-documented psychometric properties including sound reliability, and it is widely considered the gold standard instrument for assessing anxiety [30]. Within the present study, anxious youth were required to meet criteria for at least one current anxiety disorder diagnosis (CSR ≥4); youth who received sub-threshold ratings for anxiety (that is, a CSR ≤3) or who had a history of anxiety but did not currently meet criteria for a DSM-IV anxiety disorder diagnosis were excluded from participating in either arm of the study.

#### Multidimensional anxiety scale for children (MASC)

All participants completed the MASC [31], a widely used and psychometrically sound measure that assesses anxiety symptoms [2]. ANX participants had significantly higher MASC total scores compared to HC (*F* (1,30) =9.25), *P* <0.005). On the subscales, the ANX had significantly higher scores *versus* HC for harm avoidance (*F* (1,30) =4.28), *P*=0.04) and social anxiety (*F* (1,30) =6.07), *P*=0.02); there was a trend for physical symptoms (*F* (1,30)=3.5), *P*=0.07; Table 1).

### MRI data acquisition

Imaging data were collected on a 3 T Siemens Trio MRI scanner. For each run, 182 functional T2*-weighted echoplanar images (EPI) were acquired (slice thickness, 4 mm; 34 slices; TR, 2 s; TE, 30 ms; flip angle, 90°; matrix, 64 × 64; field of view (FOV), 200 mm; voxel size, 3 × 3 × 4 mm^3^]. Four volumes, collected at the beginning of each run to allow for T1 equilibrium effects, were discarded. A T2-weighted, matched-bandwidth (MBW) and high-resolution, anatomical scan and magnetization-prepared rapid-acquisition gradient echo (MPRAGE) scan were acquired for each subject for registration (TR, 2.3 s; TE, 2.1 ms; FOV, 256 mm; matrix, 192 × 192; sagittal plane; slice thickness, 1 mm; 160 slices).

After the scan, participants completed a rating scale about the scanning procedure ranging from 1 (‘the scanner was not scary at all’) to 4 (‘the scanner was very scary. I could not wait to get out’). There were no group differences on this scale (*F* (1,30)=1.9, *P*>0.5) and ratings in both the AG (*M=*1.82, *SD=*.72) and the HC (*M=*1.53, *SD=*.66) groups were low.

### Image preprocessing and registration

Imaging data were analyzed using the FSL 4.1.6 toolbox. Images were realigned to compensate for small head movements. All data reported are from scans that exhibited ≤2 mm in movement. There were no group differences in motion (AG: *M*=.12 mm; HC*: M*=.30 mm). The data were smoothed using a 5-mm FWHM Gaussian kernel, and filtered in the temporal domain using a non-linear high-pass filter (66-s cutoff). EPI images were first registered to the MBW scan, then to the MPRAGE scan, and finally into standard MNI space (MNI152, T1 2 mm) for group analyses.

The following events were modeled: neutral vignette, anxiety-provoking vignette, neutral face, and anxiety-provoking face. Events were modeled at the time of the stimulus presentation with 5 s and 1 s duration for vignettes and faces, respectively. Temporal derivatives were included as covariates of no interest to improve statistical sensitivity. Null events were not explicitly modeled and therefore constituted an implicit baseline.

As we were most interested in how social context modulates interpretations of a neutral social stimulus, we focused on analyses of face presentation but not vignette presentation. Only neutral faces were presented in this study but half were paired with a neutral vignette and half were paired with an anxiety-provoking vignette. For each participant, the following four contrasts were computed: (1) neutral-paired face - baseline; (2) anxiety-paired face - baseline; (3) neutral-paired face - anxiety-paired face; and (4) anxiety-paired face - neutral-paired face. Statistical modeling was first performed separately for each imaging run. Regressors of interest were created by convolving a *delta* function representing trial onset times with a canonical (double-gamma) hemodynamic response function. The six movement parameters that were obtained during realignment showed that rotation and translation movement within each subject and session was <2 mm in all participants; nonetheless, the movement parameters were modeled as regressors of no interest at the first level. A second-level, fixed effects analysis combined runs for each participant. A 2 (group) × 2 (vignette) × 2 (choice) repeated measures Analysis of Variance (ANOVA) was conducted at the group level using the FMRIB Local Analysis of Mixed Effects (FLAME1) module in FSL [31-33]. Z (Gaussianised T) statistic images were thresholded using clusters determined by Z >2.3 and a (whole-brain corrected) cluster significance threshold of *P* <0.05 using Gaussian Random Fields theory [34]. Tests were corrected for family-wise errors (FWE).

To examine correlations between behavioral measures and neural activity, variables of interest were modeled as explanatory variables at the third-level. For regression analyses, the outlier rejection tool in FSL (automatic outlier de-weighting) was used. This tool automatically detects outlier data points (for each voxel, each subject’s data are considered with respect to the other subjects to determine if it is an outlier) [35]. Outliers are then automatically de-weighted in the multi-subject statistics. Anatomical localization within each cluster was obtained by searching within maximum likelihood regions from the FSL Harvard-Oxford probabilistic atlas. All fMRI data shown were cluster-corrected for multiple comparison at z=2.3, *P* <0.05. For visualization purposes, percent MR signal change for regions that showed significant correlations with behavioral variables of interest were extracted and plotted against the behavioral measures.

## Results

### Behavioral results

A 2 (group) × 2 (vignette) × 2 (choice) repeated measures analysis of variance (ANOVA) was conducted to examine post-scan ratings of the vignettes. There was a significant main effect of vignette type on ratings F (1,30) =8.54, *P*=0.007 (*M*_*anxiety-provoking*_*=*1.57 (range, 0–6.5); *M*_*neutral*_*=*0.81 (range, 0–2.3)) such that the anxiety-provoking vignettes elicited greater feelings of anxiety. There were no significant effects of group or interactions. There were no significant effects of reaction time, as determined by ANOVA. After the task, participants were presented with the neutral faces and asked to provide a rating of how anxiety-provoking they were (1=not anxiety-provoking, 4=very anxiety-provoking); there was a trend towards a group difference [F(1,30)=3.02, *P*=0.06] with the anxious group (*M=*2.04) rating the faces as more anxiety-provoking than the control group (*M=*1.65).

### fMRI results

**Figure 2:**
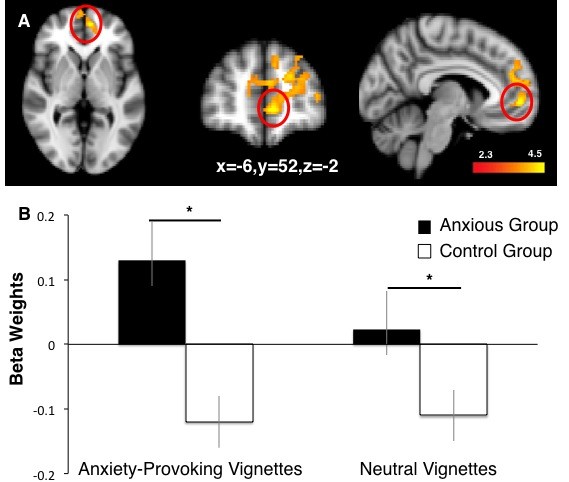
**Neural activation in anxious and healthy youth. (A)** Greater medial prefrontal cortex (mPFC) activation in response to neutral faces that had been paired with anxiety-provoking *versus* neutral contexts. **(B)** Parameter estimates from the mPFC (circled in **A**) illustrate greater activation in anxious *versus* control participants.

A repeated measures ANOVA revealed a main effect of group and interaction on activation in the mPFC (x=−6, x=52, z=−2) (z=4.34) (Figure 2A). The anxious group showed significantly greater activation to faces paired with anxiety-provoking vignettes (*M=.13)* relative to the control group (*M=−0.12*) [t(31)=4.65, *P*=0.03] and relative to faces paired with neutral vignettes (*M=0.02*) [t(31)=2.89, *P* <0.05]; the control group significantly differed from baseline in both conditions (*P* <0.05) (Figure 2B). Greater activation in mPFC was significantly correlated with ratings of the anxiety-provoking vignettes in the anxious group (*r=*0.56, *P*=0.03) but not in the control group (*r=*0.01, *ns*). mPFC activation was not correlated with neutral ratings for either group (anxious group: *r=*0.33, *ns;* control group: *r=*0.04, *ns*).

## Discussion

This study is among the first to examine how anxious youth and their non-anxious counterparts differ in their neural responses to neutral stimuli framed by different contextual cues. As predicted, the two groups were differentiated by their mPFC responses, with anxious youth showing significantly greater activation to faces paired with anxiety-provoking vignettes than control youth and minimal activation to faces paired with neutral vignettes. The control group showed no difference in activation to the two conditions. Counter to expectations, the performance/evaluation frames were not associated with amygdala reactivity for either group. We speculate that perhaps this null finding was due to the fact that the participant was an observer of the anxiety-provoking event so it was not a direct threat to them.

Efforts to understand the etiology and course of youth anxiety disorders have long emphasized the role of interpretation biases in eliciting and maintaining anxiety [9,36]. These biases include the tendency to interpret neutral stimuli as dangerous, and are thought to fuel the distorted thinking and avoidance behavior that characterize anxiety disorders. This study builds on existing work to highlight the role of the mPFC in contributing to interpretation biases for anxious youth. When viewing the same neutral faces paired with different contextual cues, anxious adolescents exhibited heightened mPFC responding to the frames that involved performance and/or evaluation, a pattern that was distinct from their non-anxious counterparts.

These findings parallel earlier work with healthy adults demonstrating that neural responses to threat are sensitive to changes in context and that these changes may modulate patterns of neural responding [24]. The role of the mPFC is of particular interest given prior research implicating it in mentalizing tasks (that is, those that require the subject to infer what another person is feeling [37]) and anxiety [19-21]. In the present investigation, anxious youth may have been more sensitive to the feelings that they associated with the performance/evaluation frames and inferred those feelings as present for the individuals depicted on the stimuli.

Interestingly, we did not find the expected group differences in amygdala reactivity. Given its established role as a threat sensor in anxious youth (see Pine et al. [38] for review), it is possible that the task involved in this study was not viewed by subjects as threatening. Indeed, the stimuli presented in this study involved neutral faces that were not designed to be evocative. Certainly, the absence of findings among healthy controls is in keeping with earlier work demonstrating that comparison subjects do not show amygdala activity when passively viewing fearful faces [39].

The present results should be interpreted in light of several study limitations. First, the sample employed in this study was small and further replication is needed. Second, anxious youth were identified based on the presence of a primary anxiety disorder and were excluded if any other co-morbid conditions were present. While this bolsters confidence that the present findings are specific to youth with anxiety disorders, the vast majority of children and adolescents with anxiety disorders present with more than one mental health condition, which may limit generalization of these findings. Finally, while our focus was on adolescents, for purposes of this pilot work, adolescence was defined broadly and the sample included a broad age range.

## Conclusions

These limitations notwithstanding, the present investigation is among the first to demonstrate that context modulates mPFC responding to neutral stimuli among anxious youth. It takes an important step toward understanding how these youth make sense of neutral stimuli and the conditions under which they might elicit heightened patterns of activation. Future research is needed to examine more definitively the role of the mPFC in adolescent anxiety and the extent to which it may serve as a biomarker for illness. In addition, research aimed at understanding the mechanisms by which current anxiety disorder treatments serve to neutralize this pattern of responding is needed.

## Competing interests

The authors declared that they have no competing interests.

## Authors’ contributions

Both authors contributed equally to this investigation. TP and AG conceived of the study, and participated in its design and coordination and helped to draft the manuscript. TP conducted all diagnostic assessments; AG oversaw all imaging procedures. Both authors read and approved the final manuscript.
